# Geographical and Seasonal Thermal Sensitivity of Grazing Pressure by Microzooplankton in Contrasting Marine Ecosystems

**DOI:** 10.3389/fmicb.2021.679863

**Published:** 2021-07-05

**Authors:** Marco J. Cabrerizo, Emilio Marañón

**Affiliations:** ^1^Centro de Investigación Mariña, Universidade de Vigo, Vigo, Spain; ^2^Departamento de Ecología y Biología Animal, Facultad de Ciencias del Mar, Universidade de Vigo, Vigo, Spain

**Keywords:** activation energy, herbivorous grazing, phytoplankton growth, plankton communities, polar ecosystems, temperate ecosystems, temperature, tropical ecosystems

## Abstract

Grazing pressure, estimated as the ratio between microzooplankton grazing and phytoplankton growth rates (*g*:μ), is a strong determinant of microbial food-web structure and element cycling in the upper ocean. It is generally accepted that *g* is more sensitive to temperature than μ, but it remains unknown how the thermal dependence (activation energy, *E*_a_) of *g*:μ varies over spatial and temporal scales. To tackle this uncertainty, we used an extensive literature analysis obtaining 751 paired rate estimates of μ and *g* from dilution experiments performed throughout the world’s marine environments. On a geographical scale, we found a stimulatory effect of temperature in polar open-ocean (∼0.5 eV) and tropical coastal (∼0.2 eV) regions, and an inhibitory one in the remaining biomes (values between −0.1 and −0.4 eV). On a seasonal scale, the temperature effect on *g*:μ ratios was stimulatory, particularly in polar environments; however, the large variability existing between estimates resulted in non-significant differences among biomes. We observed that increases in nitrate availability stimulated the temperature dependence of grazing pressure (i.e., led to more positive *E*_a_ of *g*:μ) in open-ocean ecosystems and inhibited it in coastal ones, particularly in polar environments. The percentage of primary production grazed by microzooplankton (∼56%) was similar in all regions. Our results suggest that warming of surface ocean waters could exert a highly variable impact, in terms of both magnitude and direction (stimulation or inhibition), on microzooplankton grazing pressure in different ocean regions.

## Introduction

Temperature is a key environmental driver controlling the rates at which energy and materials flow through food webs and ecosystems (Gillooly et al., 2001; Cross et al., 2015). Predictions by the metabolic theory of ecology (MTE, Brown et al., 2004) and evidence from terrestrial (Allen et al., 2005; Allen and Gillooly, 2009) and aquatic (López-Urrutia et al., 2006; Liu et al., 2019; Barton et al., 2020) ecosystems show that the thermal dependence [or activation energy (*E*_a_)] of heterotrophic processes [e.g., grazing rates (*g*)] is higher than that of autotrophic ones [e.g., phytoplankton growth rates (μ)]. A weaker thermal sensitivity of phytoplankton growth compared with microzooplankton growth and grazing activity could contribute to trigger phytoplankton blooms in cold waters (Rose and Caron, 2007). However, growing empirical evidence has shown that autotrophic processes can be as sensitive to temperature as heterotrophic ones (Chen and Laws, 2017; Wang et al., 2019). Recent experimental studies also show that the thermal dependence of phytoplankton growth and microzooplankton grazing can change seasonally, with *E*_a_ of μ exhibiting variations two- to four-fold larger than those found in *E*_a_ of *g* (Liu et al., 2019). In light of these findings, two hypotheses can be postulated. According to MTE, if *E*_a_ of *g* is higher than that of μ, *E*_a_ of *g*:μ should be positive, i.e., enhanced grazing pressure with increasing temperature. By contrast, following the arguments of Wang et al. (2019), if *E*_a_ of *g* and μ are similar, grazing pressure should be independent of temperature. In addition, the temperature dependence of plankton metabolism can be weaker (Liu et al., 2021b) and even suppressed (O’Connor et al., 2009; Marañón et al., 2018) when nutrient availability is low. Under this premise, if nutrient limitation decreases *E*_a_ of μ (without altering *E*_a_ of *g*), we could expect a lower *E*_a_ of *g*:μ in nutrient-rich, coastal, and upwelling areas than in the open ocean, where nutrient limitation of phytoplankton growth is most prevailing (Moore et al., 2013).

Despite the well-recognized ecological role of grazing pressure by microzooplankton, which represents a consumption of ∼60% of global marine primary production (Schmoker et al., 2013), most studies evaluating its sensitivity to temperature have considered microzooplankton grazing and phytoplankton growth separately (Liu et al., 2019). However, the *g*:μ ratio is a key variable to evaluate how warming could impact microbial trophic interactions because it gives a more integrative view of trophic functioning than considering both rates separately and has major implications for the fate of newly produced organic matter. Moreover, earlier studies have evaluated mostly geographical variability (Calbet and Landry, 2004; Chen et al., 2012; Liu et al., 2019), whereas changes over time have received less attention (Lawrence and Menden-Deuer, 2012; Franzè and Lavrentyev, 2014; López-Ábate et al., 2016). The importance of temporal dynamics has been stressed by Anderson and Harvey (2019), who proposed that μ and *g* should be measured over annual cycles at weekly to monthly intervals and across ecosystems to provide more accurate predictions of the fate of primary production (and related food web processes) and to ascertain the factors that drive plankton dynamics in contrasting marine regions. In addition, Li et al. (2013) have shown the feasibility of space-for-time substitution for inferring the future structural and compositional state of plankton communities in a given ecosystem from present observations across geographical gradients.

Here, we address the temperature dependence of *g*:μ in surface natural planktonic communities considering both geographical (i.e., from temperate to polar biomes in coastal and open-ocean ecosystems) and seasonal variability. We compiled a global ocean database using published reports of experimental determinations of μ and *g* using the dilution or modified dilution method (Landry and Hassett, 1982; Chen, 2015). Our main goal was to quantify the thermal sensitivity of *g*:μ over geographical and seasonal scales of variability and to assess how its magnitude and direction can be affected by changes in nutrient availability.

## Materials and Methods

### Literature Database

We surveyed the published literature until February 4, 2020 using SCOPUS^®^ and Web of Knowledge^®^ with the following keywords and Boolean operators in advanced searches: (“grazing^∗^” OR “growth^∗^”) AND (“microzooplankton^∗^” OR “phytoplankton^∗^”) AND (“seasonal variations”) and retrieved a total of 163 studies published from 1988 to 2020. After a detailed inspection of the abstracts, results, and supplementary information, when supplied, we found that 64 studies reported *in situ* μ and *g* rates using the dilution method in coastal and open-ocean ecosystems from polar, temperature and tropical biomes worldwide and 20 that reported *in situ* μ and *g* rates from the same sampling point/area over time. Additionally, we used *in situ* phytoplankton μ and microzooplankton *g* rates from an updated database made by Sherman et al. (2016) from the work by Calbet and Landry (2004).

We conducted a subsequent filtering of the data of μ and *g* retrieved, whereby data were excluded in the following cases: (1) the linear regression fit between dilution factor and phytoplankton growth rate had a determination coefficient <0.35 (Sherman et al., 2016); (2) growth rates had values of zero, which results in a value of infinite for *g*:μ; (3) rates in which μ was higher in undiluted treatments than in diluted ones; (4) experiments in which inorganic nutrients were added to the incubation bottles, and (5) experiments that did not expose samples to *in situ* temperature and light conditions. We focused our study in rates measured under non-enriched conditions because experiments where nutrients were added did not follow a consistent pattern. They differed in chemical forms applied (e.g., nitrate vs. ammonium), concentrations used (nitrate ranging between 0 and 10 μM; phosphate ranging between 0 and 1 μM), and also in the ratios N:P applied. In some cases, other macronutrients (e.g., silicate) and even different micronutrients were also added.

Additionally, and to minimize the potential effect of differences in light availability among sites and experiments, we only considered surface or near-surface waters (<30 m depth). The biomes considered (i.e., tropical, temperate, polar) were established following a latitudinal range (0–29.99°, 30–59.99°, and 60–90°, respectively) from the latitude coordinates given in the studies considered, whereas geographical areas followed the criteria already established by Sherman et al. (2016): open ocean are oceanic habitats, and coastal areas are habitats overlying the continental shelf. The rationale for assessing changes in *g*:μ ratios for each biome separately was testing how natural plankton communities with *a priori* different thermal histories respond to changes in temperature.

With the abovementioned considerations, we obtained a total of 473 (*n* polar = 83, *n* temperate = 175, *n* tropical = 215) and 278 (*n* polar = 53, *n* temperate = 113, *n* tropical = 112) valid individual data points for the geographical and seasonal analyses, respectively (Supplementary Figures 1, 2). Additional information compiled was geographical location of the sampling points (latitude and longitude), chlorophyll *a* concentration (Supplementary Figures 3A, 4A and Supplementary Tables 1, 2), nitrate concentration (Supplementary Figures 3B, 4B and Supplementary Tables 2, 3), and temperature (°C). Considering the scarcity of data on nutrients other than NO_3_^–^, and the fact that nitrogen is the primary limiting nutrient in the majority of the ocean (Moore et al., 2013), we focused on NO_3_^–^ availability to assess the role of nutrient limitation.

### Statistical Analysis

From the two original databases, data were divided to study grazing pressure response, as *g*:μ ratios, in global open-ocean versus coastal ecosystems, in polar, tropical, and temperate ecosystems, as well as in polar, tropical, and temperate open-ocean and coastal ecosystems. By considering temperature as predictor variable, and *g*:μ as dependent variable, we used ordinary least-squares regression to determine the apparent activation energy of grazing pressure, i.e., the slope (−*E*_a_) of the linear relationship between 1/*K*T and the natural logarithm of the *g*:μ *ratio*, where *K* is the Boltzmann’s constant (8.62 × 10^–5^ eV K^–1^) and *T* is temperature in *K*. We used temperature as a single predictor variable because previous stepwise multiple linear regression analyses (with temperature, nitrate and Chl *a* as predictors) evidenced that it was the only significant variable (data not shown). In the geographical analysis, some *g*:μ ratios had extremely low values, which were identified as outliers after calculating the lower inner fences using the formula Q1–1.5 × IQR where Q1 is the 25th percentile and IQR is the interquartile range (difference between the 75th and 25th percentiles). Therefore, we tested how the exclusion of such values could affect the slope of the linear fit model and thus the response pattern observed. Since in no case did we find significant differences between models (Supplementary Figure 5 and Supplementary Table 4), we used the slope values obtained after extremely low *g*:μ data points were excluded. Linear regression models were fitted to assess the relationship between apparent *E*_a_ and NO_3_^–^ availability in the geographical and seasonal approaches. Two-way analysis of the variance (ANOVA) was used to test for interactions between area (i.e., coastal vs. open-ocean) and biome type (i.e., polar/boreal, temperate and tropical) on apparent *E*_a_ of *g*:μ ratio for the geographical study. Also, two-way ANOVA, with study (i.e., this study vs. Calbet and Landry, 2004) and biome or area type (i.e., global comparisons, open-ocean vs. coastal comparisons, and temperate, tropical, and boreal/polar) as factors, were used to test for significant differences between estimates of *g*:μ ratios. One-way ANOVA, with biome type as factor, was used to test for significant differences in the apparent *E*_a_ of *g*:μ ratio quantified in the seasonal approach. Before performing regression and ANOVA analyses, assumptions of homogeneity of variance (by Shapiro-Wilk’s test and by residual vs. fitted values plots for linear regression) and normal distribution (by Kolmogorov-Smirnov’s test) of the errors, and independence of the predictor variable respect to the explanatory variable (by Pearson’s correlation coefficient) were checked. Because such assumptions were not met for apparent *E*_a_ in the seasonal approach, original data were 1/*x*-transformed. When interactions were significant, a Least Significant Differences (LSD) *post hoc* test was performed. Student’s *t*-test was used to evaluate significant differences between apparent *E*_a_ values obtained in the geographical analysis using the full dataset and dataset with low *g*:μ ratios excluded. Mann–Whitney *U* test was used to test for significant differences in NO_3_^–^ and Chl *a* concentrations among biomes and/or within areas in both approaches.

## Results

### Chl a Concentrations on a Geographical vs. Seasonal Approach

Chl *a* concentrations showed low median values (<2 μg L^–1^) regardless of the ecosystem considered; however, such values ranged between ∼0.1 and ca. 18 μg L^–1^ (temperate_coast_), and significant differences were found between most biomes and areas considered (Supplementary Figure 3A and Supplementary Table 1). For the seasonal approach, we also found significant differences in Chl *a* concentration between biomes, with median values being lower in polar (i.e., 1.35 μg L^–1^) than in temperate (3.90 μg L^–1^) and tropical (5.60 μg L^–1^) regions (Supplementary Figure 4A and Supplementary Table 2). The higher Chl *a* concentration observed in the tropical biome was likely due to a higher contribution of samples from coastal waters (e.g., estuaries and lagoons).

### Nitrate Concentrations on a Geographical vs. Seasonal Approach

Median nitrate concentrations were <1 μM in tropical ecosystems, 3.10–5.40 μM in temperate regions, and 6–21 μM in polar ones. These concentrations were significantly different between coastal and open-ocean areas in polar (*Z* = −3.76, *p* < 0.001) and temperate (Z = 2.28, *p* < 0.05) biomes, but not in tropical ecosystems (Z = −0.60, *p* = 0.55) (Supplementary Figure 3B and Supplementary Table 3). Nitrate concentrations measured in biomes considered in the seasonal analysis were significantly higher in polar (i.e., ∼10 μM) compared with tropical (i.e., 1.40 μM) and temperate (i.e., 0.80 μM) ones (Supplementary Figure 4B and Supplementary Table 2).

### Grazing Pressure by Microzooplankton on a Geographical vs. Seasonal Approach

**FIGURE 1:**
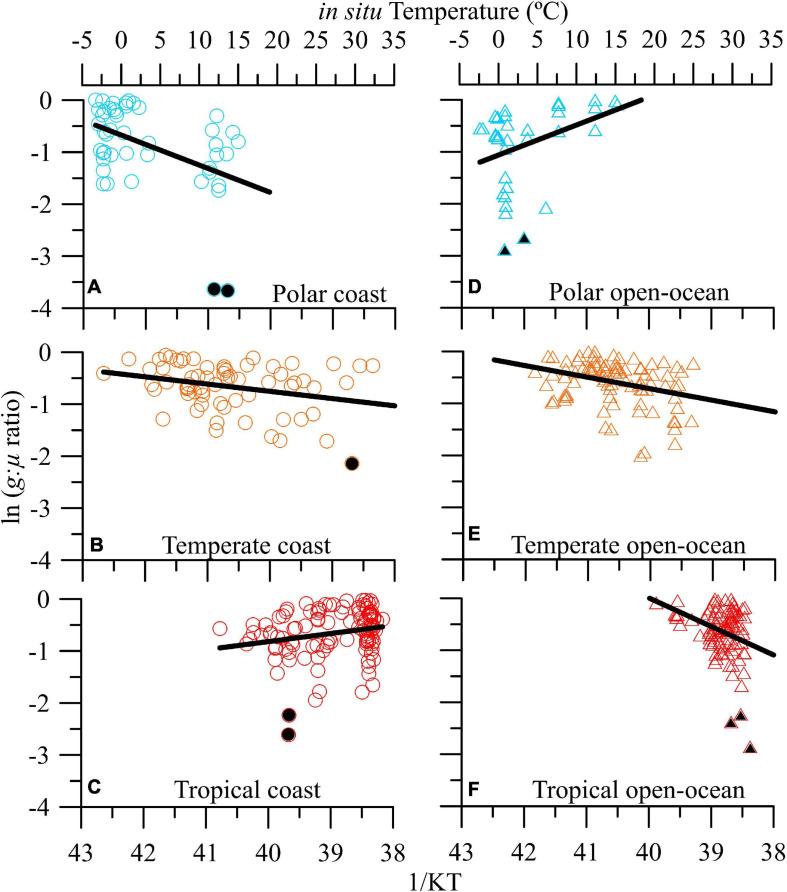
Geographical analysis. Natural logarithm of microzooplankton grazing:phytoplankton growth ratio (*g*:μ) versus *in situ* temperature in natural plankton communities from coastal **(A–C)** and open-ocean **(D–F)** areas in polar, temperate, and tropical biomes. Solid lines denote the linear regression fit. Data points indicated with filled symbols were excluded from the linear regression analyses (see section “Materials and Methods”).

Global ocean-scale ln *g*:μ ratios ranged between ∼0 and -3.70 (which corresponds to ca. 0.02–1 in non-transformed values; Supplementary Figure 2A). The strongest grazing pressure was similar in coastal and open ocean ecosystems (i.e., values ∼0, which correspond to *g*:μ ratios near 1), whereas the weakest was observed in open-ocean ecosystems (i.e., -3.66, or a non-transformed *g*:μ ratio of 0.06; Supplementary Figures 2B,C). No significant relationship was found between temperature (which ranged from ca. −3 to 31°C) and *g*:μ ratios, neither when all ecosystems were pooled together nor when we differentiated them by areas (i.e., coastal vs. open-ocean). However, at the biome level, there was a modest but significant negative thermal dependence of *g*:μ ratios for temperate ecosystems (*E*_a_ = -0.10 ± 0.04; *R*^2^ = 0.05, *F*_166_ = 5.23, *p* < 0.01; Supplementary Figure 2E). When we performed a detailed geographical analysis, distinguishing between areas and biome types (i.e., polar/boreal, temperature, and tropical), two response patterns emerged: (1) a negative relationship between temperature and *g*:μ ratios, observed in polar and temperate coastal ecosystems (Figures 1A,B and Supplementary Table 5) as well as in temperate and tropical open-ocean ones (Figures 1E,F and Supplementary Table 5), and (2) a positive relationship between temperature and grazing pressure, as found in tropical coastal and polar open-ocean ecosystems (Figures 1C,D and Supplementary Table 5).

**FIGURE 2:**
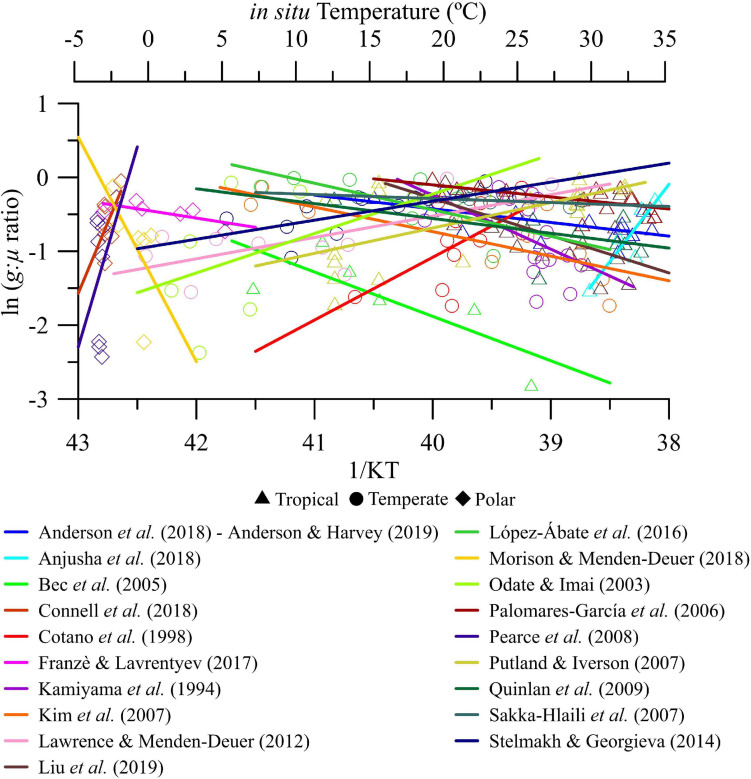
Seasonal analysis. Natural logarithm of microzooplankton grazing:phytoplankton growth ratio (*g*:μ) versus *in situ* temperature in natural plankton communities coming from polar, temperate, and tropical biomes. Note that each individually colored solid line represents the linear regression fit of an experimental study performed in a specific coastal study site over time.

Over the seasonal scale of variability, *g*:μ ratios ranged between ∼0 and −3 (or between 0.05 and ∼1 in non-transformed values) over the thermal gradient considered (Figure 2). Also, we found that the magnitude (i.e., slope absolute values) and direction (i.e., positive or negative) of the temperature effect on *g*:μ ratios was highly variable regardless of the biome (Figure 2 and Supplementary Table 6).

### Thermal Sensitivity of Grazing Pressure by Microzooplankton: Geographical vs. Seasonal Patterns

**FIGURE 3:**
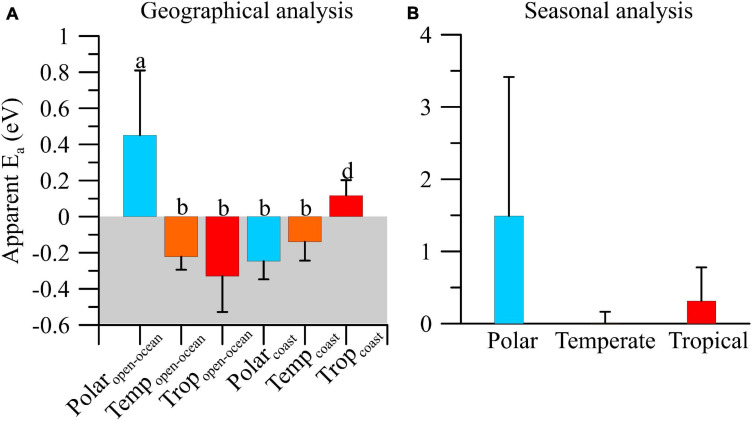
Mean (±SE) apparent activation energy (*E*_a_) of microzooplankton grazing:phytoplankton growth ratio (*g*:μ) on a geographical **(A)** and seasonal **(B)** analyses in polar, temperate, and tropical biomes.

**FIGURE 4:**
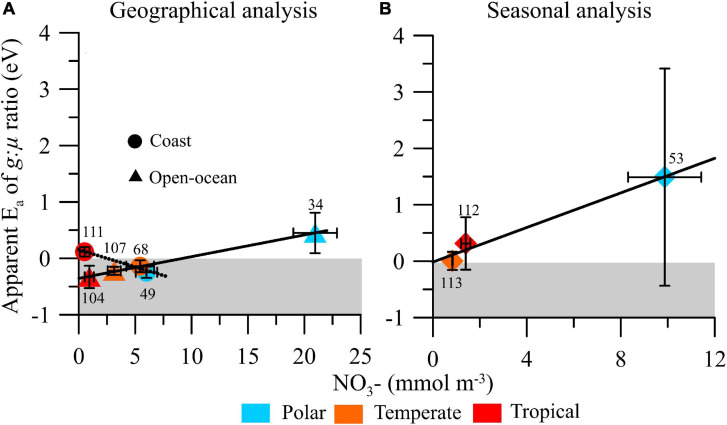
Mean (±SE) apparent activation energy (*E*_a_) of microzooplankton grazing:phytoplankton growth ratio (*g*:μ) on a **(A)** geographical and **(B)** seasonal analysis in polar, temperate, and tropical biomes and their relationship with median (±SE) nitrate availability (in mmol NO_3_^–^ m^–3^). Solid and dashed lines represent the fitted linear regression models, and numbers next to the symbols indicate the number of estimates obtained for each biome/area.

From *g*:μ ratios and temperature we calculated the apparent *E*_a_ as shown in Figures 3, 4. Apparent *E*_a_ values indicated a variable thermal dependence of *g*:μ ratios due to a significant biome × area interacting effect (*df* = 2, *F* = 15.24, *p* < 0.001; Figure 3). Temperature exerted a stimulatory effect on *g*:μ ratio (i.e., increased grazing pressure) in polar open-ocean (∼0.5 eV) and, to a lesser extent, tropical coastal (∼0.2 eV) ecosystems (Figure 3A). By contrast, increasing temperature resulted in decreased grazing pressure in the remaining areas and biomes. Specifically, rising temperature exerted a stronger inhibitory effect in tropical open-ocean ecosystems (*E*_a_ ∼−0.4 eV) followed by those on polar coastal and temperate ecosystems, where no significant differences between areas were detected (*E*_a_ ∼−0.1 and −0.2 eV) (Figure 3A). Considering the seasonal variability, apparent *E*_a_ values showed that the overall mean temperature effect on *g*:μ ratios was slightly stimulatory, particularly in polar environments; however, due to the large variability existing we did not find significant differences among biomes (*df* = 2, *F* = 0.05, *p* = ∼0.95; Figure 3B).

### Thermal Sensitivity of Grazing Pressure by Microzooplankton: Effect of Nitrate Availability

We also explored the relationship between nitrate availability and apparent *E*_a_ of *g*:μ (Figure 4). For the geographical analysis, we found significant and opposite response patterns in open-ocean [*y* = 0.04 × −0.35, *R*^2^ = 0.80, *p* = 0.0024] and coastal [*y* = −0.06 × + 0.15, *R*^2^ = 0.76, *p* = 0.0023] ecosystems, with increasing nitrate availability stimulating (i.e., leading to more positive *E*_a_) the temperature dependence of *g*:μ in open-ocean ecosystems and weakening it in coastal ones (Figure 4A). In the seasonal analysis, we found a positive but not significant relationship between the temperature sensitivity of *g*:μ ratio and nitrate availability ([*y* = 0.15 × −0.02, *R*^2^ = 0.31, *p* = 0.1180; Figure 4B).

### Recent vs. Previous Estimates of Grazing Pressure by Microzooplankton in the Ocean

**FIGURE 5:**
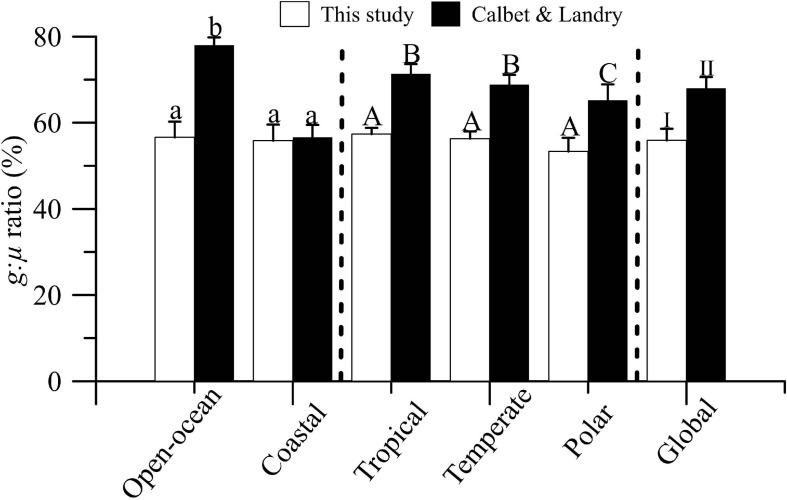
Mean (±SE) microzooplankton grazing:phytoplankton growth ratios (*g*:μ,%) in global ocean, open-ocean vs. coastal ecosystems, and temperate, tropical, and polar/boreal biomes. Solid bars represent estimates extracted from Table 2 (reduced data) of Calbet and Landry (2004). Letters and numbers on top of bars represent significant differences by LSD *post hoc* test.

Finally, we compared our *g*:μ estimates with previous results by Calbet and Landry (2004; Figure 5). Although our database included a ∼50% of new estimates (i.e., 219 estimates), we found that *g*:μ ratios, i.e., the percentage of primary production grazed by microzooplankton, was similar in the two studies, regardless of the biome or region considered. However, in our dataset, microzooplankton grazing represented, on average, 56% of phytoplankton production, compared with ∼68% found by Calbet and Landry (Table 2; reduced dataset).

## Discussion

Our results show that the temperature dependence of grazing pressure is highly variable among biomes and regions. This high variability is particularly marked (up to three-fold higher) when the thermal dependence is addressed over a seasonal instead of a geographical scale. In addition, we found that even within the same biome (i.e., polar), rising temperature can exert a dual effect, accentuating or reducing grazing pressure. These patterns differ from predictions by MTE (Brown et al., 2004; Bruno et al., 2015) and field observations supporting an increased grazing pressure with rising temperature (O’Connor et al., 2009; Chen et al., 2012; Carr et al., 2018; Liu et al., 2021a,b). By contrast, our results align with experimental evidence showing a negative relationship between temperature and grazing pressure (Menden-Deuer et al., 2018; Liu et al., 2019). Although the latter studies considered only short-term and transient responses to abrupt temperature changes, recent findings have shown that lack of acclimation do not necessarily affect microzooplankton activity (Franzè and Menden-Deuer, 2020).

According to the MTE, the different *E*_a_ of autotrophic versus heterotrophic metabolic rates arises because RUBISCO has a lower thermal sensitivity than processes associated with heterotrophy (Allen et al., 2005). However, these predictions are based on land plant ecophysiology and do not consider some particular characteristics of photosynthetic unicells (i.e., presence of inorganic carbon concentrating mechanisms, Raven et al., 2008), and additional constraints such as the different availability of CO_2_ in water and air and the different solubility of CO_2_ and O_2_ (Sarmiento and Gruber, 2013). A recent study by Wang et al. (2019) challenging this paradigm proposes that the thermal sensitivity of autotroph growth rates can be as high as that of heterotroph growth when considering within-taxon responses. In addition, considering that gross growth efficiency is relatively constrained in microzooplankton (∼30%; Straile, 1997) which means that the coupling between grazing and growth is high, it is likely that both rates exhibit comparable thermal sensitivity. A similar *E*_a_ for both phytoplankton growth and microzooplankton grazing would explain the absence of thermal dependence of grazing pressure (*g*:μ ratio) reported by Sherman et al. (2016), which differs from the results reported here, where temperature either stimulated or inhibited grazing pressure.

One unexpected result from our geographic analysis is that grazing pressure decreased with increasing temperature in four of the six ecosystems considered. Several non-exclusive mechanisms may help explain this pattern. For instance, secondary metabolites with allelopathic effect such as polyunsaturated aldehydes (PUA), which are abundant both in coastal and open-ocean areas and oceanic gyres (Cózar et al., 2018), can be a strong deterrent for microzooplankton herbivory (Franzè et al., 2018). Compounds produced by dominant phytoplankton groups in coastal and open-ocean areas (e.g., PUAs by diatoms, microcystins-like compounds by *Synechococcus*) have been shown to inhibit microzooplankton growth in natural communities (Lavrentyev et al., 2015; Sliwinska-Wilczewska et al., 2017). These allelopathic effects can be enhanced by warming (Felpeto et al., 2019) and nutrient-limited conditions (Fistarol et al., 2005). Also, warmer environments and/or low (or limiting) nutrient availability can lead to increased phytoplankton carbon:nutrient stoichiometry, i.e., seston with reduced nutritional quality (De Senerpont Domis et al., 2014), which constitutes an effective defense mechanism against herbivore predators (Branco et al., 2020), and could help explain inverse relationship between temperature and grazing pressure found in temperate, tropical open-ocean and polar coastal ecosystems. A negative relationship between temperature and *g*:μ may have also arisen from a decrease in microzooplankton biomass with increasing temperature. In this connection, Chen et al. (2012) showed that a reduced grazing pressure with increasing temperature in oligotrophic waters was associated with a decrease in microzooplankton biomass. Moreover, global-ocean-scale findings by Chust et al. (2014) suggest that rising temperatures will accentuate these reductions in zooplankton biomass by 11%, on average, and that they will be particularly prevalent in temperate and tropical coastal and open-ocean areas. The decreased microzooplankton biomass could be due to a higher energetic demand (e.g., respiration; Clarke and Frasser, 2004) or feeding thresholds (Buitenhuis et al., 2010) imposed by warm temperatures. Finally, and because we used data from natural plankton assemblages, it is likely that different populations making up the phytoplankton assemblage were experiencing different growth phases (i.e., exponential vs. stationary) at the same time. For instance, *Synechococcus* populations in the early-mid exponential growth phase have been shown to suffer larger predation by *Oxyrrhis marina* than populations in stationary phase (Apple et al., 2011). Thus, the reduced grazing pressure reported here in some areas could be also consequence that the communities studied were dominated by phytoplankton populations in stationary growth phases.

The deviation in the thermal sensitivity of grazing pressure from MTE predictions may also arise because of other ecological (e.g., community composition, biomass availability) and environmental (e.g., nitrate concentration) factors that covary with temperature. For instance, we found that a higher nitrate availability (>7 mmol m^–3^) was associated with a stimulatory effect of temperature on grazing pressure in open-ocean areas, and an inhibitory one in coastal ones. One plausible explanation could be the above mentioned fact that low nutrient availability increases the carbon:nutrient ratios in phytoplankton, thus reducing its nutritional quality for grazers and potentially reducing losses to grazing.

To the extent that the variability observed reflects the response of organisms acclimated and adapted to local thermal conditions, the observed patterns are relevant to predict the response of marine plankton communities to ocean warming. The highly variable apparent temperature dependence of grazing pressure by microzooplankton on a geographical and seasonal scale suggests that communities inhabiting polar regions could be as sensitive to warming as those inhabiting tropical ones, hence rising temperatures will not necessarily have the strongest effect on tropical species, as traditionally thought, because their optimal temperature for growth is close to current mean temperature (Kingsolver, 2009; Thomas et al., 2012).

Our analysis covered a wide range of marine environments characterized by markedly different phytoplankton size structure [from a dominance by small cells in unproductive regions to a dominance by large cells in highly productive ones; see reviews by Chisholm (1992) and Marañón (2015)]. We found that the proportion of primary production grazed by microzooplankton in all regions is relatively similar, in line with previous ocean-scale patterns reported in regions with contrasting phytoplankton size structure (Schmoker et al., 2013). A similar grazing pressure in ecosystems with widely different size structure (coast vs. open-ocean) is at odds with the view that large cell size provides a refuge from predation (Kiørboe, 1993) and that the increased dominance of larger cells during blooms arises solely from a top-down mechanism (Irigoien et al., 2005). An alternative though not mutually exclusive mechanism is that large (e.g., 10–20 μm in diameter) cells are capable of sustaining faster maximum growth rate than their smaller counterparts (Marañón et al., 2013), thus being more successful in exploiting conditions of enhanced resource availability.

In conclusion, our study reveals a complex relationship between temperature and microzooplankton grazing pressure in marine ecosystems. Rising temperature stimulates grazing pressure by microzooplankton in polar open-ocean and tropical coastal environments, which could potentially reduce the amount of newly produced biomass available for direct transfer to upper trophic levels, thus favoring the microbial food web. On the other hand, increasing temperature is associated with decreased grazing pressure in the remaining regions, potentially favoring the herbivorous food chain. Increased nutrient availability enhances the thermal dependence of grazing pressure in open-ocean ecosystems but attenuates it in coastal ones, particularly in polar environments.

## Data Availability Statement

The original contributions presented in the study are included in the article/Supplementary Material, further inquiries can be directed to the corresponding author/s.

## Author Contributions

MJC and EM conceived and designed the research. MJC compiled the dataset, analyzed, and interpreted the data, wrote the first draft and edited the manuscript, reviewed the subsequent drafts, and approved the final version. EM analyzed and interpreted the data, reviewed the subsequent drafts, and approved the final version. Both authors contributed to the article and approved the submitted version.

## Conflict of Interest

The authors declare that the research was conducted in the absence of any commercial or financial relationships that could be construed as a potential conflict of interest.
