# Supplementary material for: Geographical and Seasonal Thermal Sensitivity of Grazing Pressure by Microzooplankton in Contrasting Marine Ecosystems
Source: Front Microbiol. 2021 Jul 5;12:679863. doi: 10.3389/fmicb.2021.679863 (PMC8287633; doi:10.3389/fmicb.2021.679863)
Supplement: Supplementary file 1 [file Table_1.DOCX]

***Supplementary material***

**1. Supplementary Figures**

**Supplementary Figure S1.** Global distribution of the sampling stations considered. Note that some points cannot be visible in the map due to their proximity with other stations. Map generated with Ocean Data View v. 4.7.4 (https://odv.awi.de).


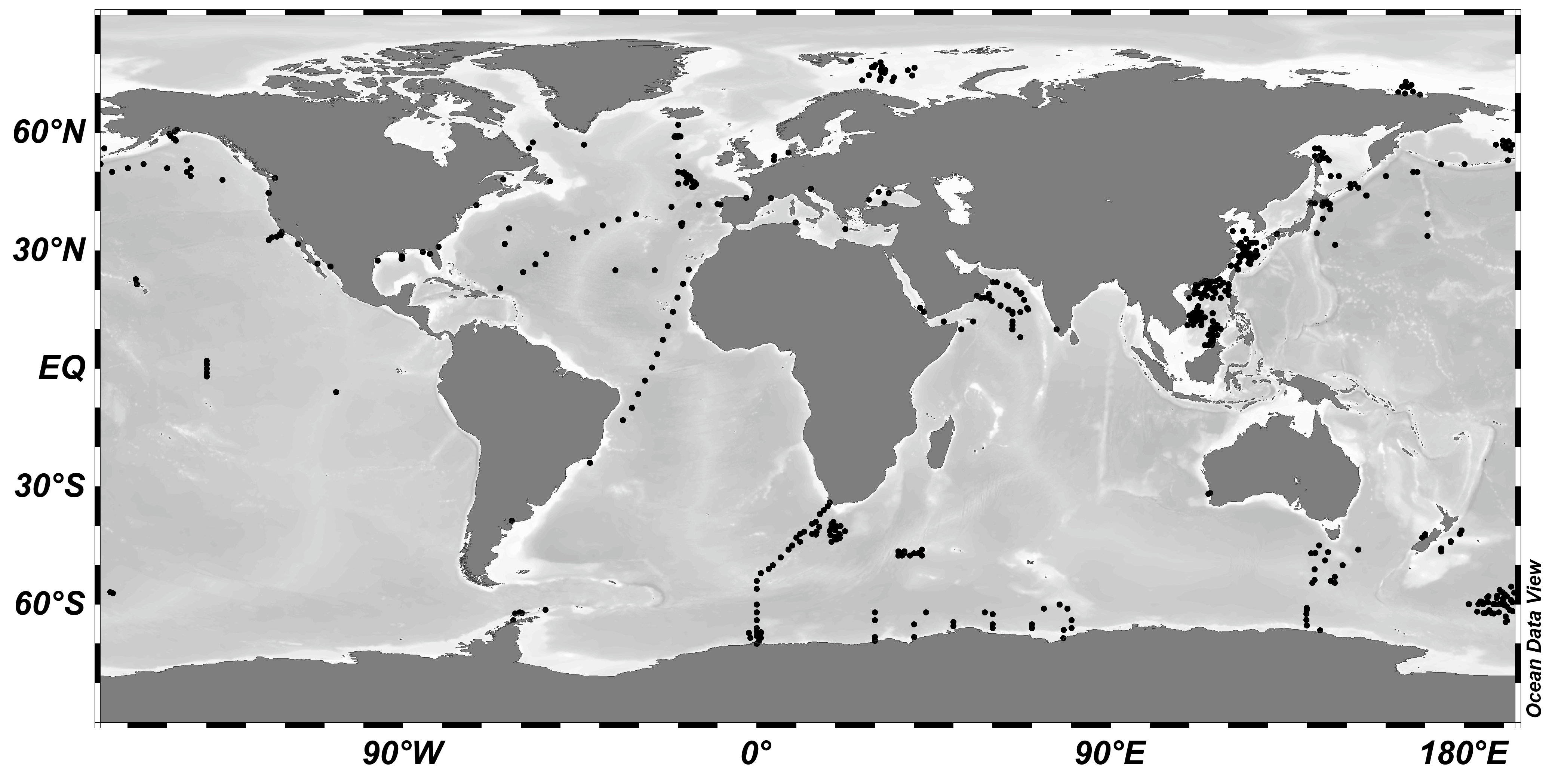


**Supplementary Figure S2.** Mean natural logarithm of microzooplankton grazing : phytoplankton growth ratio (*g:µ*) versus *in situ* temperature in natural plankton communities coming for all aquatic ecosystems pooled together (A), coastal (B) and open-ocean (C) areas, and polar (D), temperate (E) and tropical (F) biomes. Solid line denotes the linear regression fit when significant (p < 0.05).

**Supplementary Figure S3.** Box plots showing the median chlorophyll *a* (A) and nitrate (B) concentrations, together with 1.5 of the interquartile range (IQR) and outliers, in polar, temperate and tropical environments in coastal and open-ocean areas where phytoplankton growth and microzooplankton rates were measured.

**Supplementary Figure S4.** Box plots showing the median chlorophyll *a* (Chl *a*) (A) and nitrate (B) concentrations, together with 1.5 of the interquartile range (IQR) and outliers, in polar, temperate and tropical environments where phytoplankton growth and microzooplankton rates were measured in the same sampling point over time.

**Supplementary Figure S5.** Mean (±SE) apparent activation energy (E_a_) of natural logarithm of microzooplankton grazing:phytoplankton growth ratio (*g:µ*) on a geographical pattern in polar, temperate and tropical biomes. Note that white bars represent values obtained through linear regression fits from full dataset (see Fig. 1), and black bars those values obtained from the same dataset but excluding extremely low ln *g:µ* ratios (see material and methods; Fig. 1 black symbols).

**2. Supplementary Tables**

**Supplementary Table S1.** Results of the U-Mann Whitney tests for independent samples evaluating significant differences in chlorophyll *a* concentration among areas (C, coast and O, open-ocean) in polar (P), temperate (T) and tropical (Tr) biomes for the geographical analysis. Z represents Z-score, and p the p-value.

| Area/Biome | Z | p |
| --- | --- | --- |
| CP vs. CT | -1.37 | 0.17 |
| CP vs. CTr | 3.88 | < 0.0001 |
| CP vs. OP | 2.45 | < 0.01 |
| CP vs. OT | 1.53 | 0.13 |
| CP vs. OTr | 5.04 | < 0.0001 |
| CT vs. CTr | 5.51 | < 0.0001 |
| CT vs. OP | 4.48 | < 0.0001 |
| CT vs. OT | 4.51 | < 0.0001 |
| CT vs. OTr | 8.78 | < 0.0001 |
| CTr vs. OP | -2.10 | < 0.05 |
| CTr vs. OT | -4.11 | < 0.0001 |
| CTr vs. OTr | -0.47 | 0.64 |
| OP vs. OT | -1.82 | 0.07 |
| OP vs. OTr | 3.09 | < 0.01 |
| OT vs. OTr | 6.87 | < 0.0001 |

**Supplementary Table S2.** Results of the U-Mann Whitney tests for independent samples evaluating significant differences in chlorophyll *a* (Chl *a*) and nitrate concentrations among biomes in the seasonal analysis. P, T and Tr represent polar, temperate and tropical ecosystems. Z represents Z-score, and p the p-value.

|  | Chl *a* | | Nitrate | |
| --- | --- | --- | --- | --- |
| Biome | Z | p | Z | p |
| P vs. T | -9.56 | < 0.0001 | 5.14 | < 0.0001 |
| P vs. Tr | -9.17 | < 0.0001 | 2.14 | < 0.0001 |
| T vs. Tr | -8.63 | < 0.0001 | 0.00 | 0.90 |

**Supplementary Table S3.** Results of the U-Mann Whitney tests for independent samples evaluating significant differences in nitrate concentrations among areas (C, coast and O, open-ocean) in polar (P), temperate (T) and tropical (Tr) biomes for the geographical analysis. Z represents Z-score, and p the p-value.

| Area/Biome | Z | p |
| --- | --- | --- |
| CP vs. CT | -1.22 | 0.18 |
| CP vs. CTr | 3.40 | < 0.001 |
| CP vs. OP | -3.76 | < 0.001 |
| CP vs. OT | 0.88 | 0.3800 |
| CP vs. OTr | 3.19 | < 0.001 |
| CT vs. CTr | 5.55 | < 0.0001 |
| CT vs. OP | -3.08 | < 0.01 |
| CT vs. OT | 2.28 | < 0.05 |
| CT vs. OTr | 5.19 | < 0.0001 |
| CTr vs. OP | -6.61 | < 0.0001 |
| CTr vs. OT | -3.80 | < 0.0001 |
| CTr vs. OTr | -0.60 | 0.55 |
| OP vs. OT | 4.99 | < 0.0001 |
| OP vs. OTr | 6.78 | < 0.0001 |
| OT vs. OTr | 3.40 | < 0.001 |

**Supplementary Table S4.** Results of the Student’s *t*-tests evaluating significant differences in the apparent activation energy (E_a_) of natural logarithm of microzooplankton grazing:phytoplankton growth ratio (*g:µ*) on a geographical pattern in polar, temperate and tropical biomes from open-ocean and coastal areas when extremely low *g:µ* ratio values were excluded (Fig. S5, black bars) or not (Fig. S5, white bars). n.t. represents not tested because any value was excluded, and p the p-value.

| Biome_area_ | *t* | p |
| --- | --- | --- |
| Polar_open-ocean_ | 0.05 | 0.96 |
| Temperate_open-ocean_ | n.t. | n.t. |
| Tropical_open-ocean_ | -1.44 | 0.22 |
| Polar_coast_ | -2.23 | 0.10 |
| Temperate_coast_ | -0.13 | 0.90 |
| Tropical_coast_ | 0.60 | 0.58 |

**Supplementary Table S5.** Results of the ordinary least-squares regression of the thermal dependence of natural logarithm of the microzooplankton grazing rate: phytoplankton growth rate (*g:µ* ratio) in geographical experiments carried out in polar, temperature and tropical biomes from coast and open-ocean areas. R^2^ represents the determination coefficient, *F* the *F-*Snedecor test value, and p the p-value.

|  | Open-ocean | | | Coast | | |
| --- | --- | --- | --- | --- | --- | --- |
| Biome | R^2^ | *F* | p | R^2^ | *F* | p |
| Polar | 0.14 | 1.57 | < 0.001 | 0.13 | 6.02 | < 0.01 |
| Temperate | 0.11 | 9.48 | < 0.01 | 0.10 | 1.28 | < 0.01 |
| Tropical | 0.10 | 0.14 | < 0.01 | 0.07 | 1.91 | < 0.01 |

**Supplementary Table S6.** Results of the ordinary least-squares regression of the thermal dependence of natural logarithm of the microzooplankton grazing rate : phytoplankton growth rate (*g:µ* ratio) in seasonal experiments carried out in the same sampling point in polar, temperature and tropical biomes. R^2^ represents the determination coefficient, *F* the *F-*Snedecor test value, and p the p-value. Colored solid lines refer to the values and linear regression fits shown in Figure 2.

| Reference | R^2^ | *F* | p |
| --- | --- | --- | --- |
| Anderson & Harvey (2019) - Anderson *et al.* (2018) | 0.70 | 8.90 | ~0.05 |
| Anjusha *et al.* (2018) | 0.40 | 3.80 | ~0.01 |
| Bec *et al.* (2005) | 0.31 | 3.65 | ~0.01 |
| Connell *et al.* (2018) | 0.45 | 8.67 | < 0.05 |
| Cotano *et al.* (1998) | 0.32 | 3.30 | 0.14 |
| Franzè & Lavrentyev (2017) | 0.26 | 5.83 | < 0.05 |
| Kamiyama *et al.* (1994) | 0.60 | 12.53 | < 0.01 |
| Kim *et al.* (2007) | 0.20 | 4.24 | ~0.05 |
| Lawrence & Menden-Deuer (2012) | 0.33 | 8.41 | <0.01 |
| Liu *et al.* (2019) | 0.40 | 5.47 | ~0.05 |
| López-Ábate *et al.* (2016) | 0.41 | 4.35 | 0.10 |
| Morison & Menden-Deuer (2018) | 0.15 | 2.49 | 0.15 |
| Odate & Imai (2003) | 0.31 | 4.42 | 0.07 |
| Palomares-García *et al.* (2006) | 0.40 | 11.63 | < 0.01 |
| Pearce *et al.* (2008) | 0.13 | 2.04 | 0.19 |
| Putland & Iverson (2007) | 0.38 | 11.36 | < 0.01 |
| Quinlan *et al.* (2009) | 0.32 | 4.22 | 0.08 |
| Sakka-Hlaili *et al.* (2007) | 0.35 | 3.38 | 0.14 |
| Stelmakh & Georgieva (2014) | 0.57 | 14.14 | < 0.01 |

**3. Supplementary References**

Anderson, S.R., Diou-Cass, Q.P. & Harvey, E.L. (2018). Short-term estimates of phytoplankton growth and mortality in a tidal estuary. *Limnol. Oceanogr.*, 63, 2411-2422.

Anderson, S.R. & Harvey, E.L. (2019). Seasonal variability and drivers of microzooplankton grazing and phytoplankton growth in a subtropical estuary. *Front. Mar. Sci.*, 6, 174.

Anjusha, A., Jyothibabu, R., Savitha, K.M.M. & Albin, K.J. (2018). Seasonal variation of phytoplankton growth and microzooplankton grazing in a tropical coastal water (off Kochi), Southwest coast of India. *Cont. S. Res.*, 171, 12-20.

Bec, B., Husseini-Ratrema, J., Collos, Y., Souchu, P. & Vaquer, A. (2005). Phytoplankton seasonal dynamics in a Mediterranean coastal lagoon: Emphasis on the picoeukaryote community. *J. Plankton Res.*, 27, 881-894.

Connell, P.E., Michel, C., Meisterhans, G., Arrigo, K.R. & Caron, D.A. (2018). Phytoplankton and bacterial dynamics on the Chukchi Sea Shelf during the spring−summer transition. *Mar. Eco. Progr. Ser.*, 602, 49-62.

Cotano, U., Uriarte, I. & Villate, F. (1998). Herbivory of nanozooplankton in polyhaline and euhaline zones of a small temperate estuarine system (Estuary of Mundaka): Seasonal variations. *J. Exp. Mar. Biol. Ecol.*, 227, 265-279.

Franzè, G. & Lavrentyev, P.J. (2017). Microbial food web structure and dynamics across a natural temperature gradient in a productive polar shelf system. *Mar. Ecol. Progr. Ser.*, 569, 89-102.

Kamiyama, T. (1994). The impact of grazing by microzooplanktonin northern Hiroshima Bay, the Seto Inland Seam, Japan. *Mar. Biol.*, 119, 77-88.

Kim, S., Park, M.G., Moon, C., Shin, K. & Chang, M. (2007). Seasonal variations in phytoplankton growth and microzooplankton grazing in a temperate coastal embayment, Korea. *Estuar., Coast. S. Sci.* 71, 159-169.

Lawrence, C. & Menden-Deuer, S. (2012). Drivers of protistan grazing pressure: seasonal signals of plankton community composition and environmental conditions. *Mar. Ecol. Progr. Ser.*, 459, 39-52.

Liu, K., Chen, B., Zhang, S., Sato, M., Shi, Z. & Liu, H. (2019). Marine phytoplankton in subtropical coastal waters showing lower thermal sensitivity than microzooplankton. *Limnol. Oceanogr.*, 64, 1103-1119.

López-Ábate, M.C., Barría de Cao, M.S., Pettigrosso, R.E., Guinder, V.A., Dutto, M.S., Berasategui, A.A. *et al.* (2016). Seasonal changes in microzooplankton feeding behavior under varying eutrophication level in the Bahía Blanca estuary (SW Atlantic Ocean). *J. Exper. Mar. Biol. Ecol.*, 481, 25-33.

Morison, F. & Menden-Deuer, S. (2018). Seasonal similarity in rates of protistan herbivory in fjords along the Western Antarctic Peninsula. *Limnol. Oceanogr.*, 63, 2858-2876.

Odate, T. & Imai, K. (2003). Seasonal variation in chlorophyll-specific growth and microzooplankton grazing of phytoplankton in Japanese coastal water. *J. Plankton Res.*, 25, 1497-1505.

Palomares-García, R., Bustillos-Guzmán, J.J. & López-Cortés, D. (2006). Pigment-specific rates of phytoplankton growth and microzooplankton grazing in a subtropical lagoon. *J. Plankton Res.*, 28, 1217-1232.

Pearce, I., Davidson, A.T., Wright, S. & van den Enden, R. (2008). Seasonal changes in phytoplankton growth and microzooplankton grazing at an Antarctic coastal site. *Aquat. Microb. Ecol.*, 50, 157-167.

Putland, J.N. & Iverson, R.L. (2007). Microzooplankton: Major herbivores in an estuarine planktonic food web. *Mar. Ecol. Progr. Ser.*, 345, 63-73.

Quinlan, E.L., Jett, C.H. & Phlips, E.J. (2009). Microzooplankton grazing and the control of phytoplankton biomass in the Suwannee River estuary, USA. *Hydrobiologia*, 632, 127-137.

Sakka-Hlaili, A., Grami, B., Mabrouk, H.H., Gosselin, M. & Hamel, D. (2007). Phytoplankton growth and microzooplankton grazing rates in a restricted Mediterranean lagoon (Bizerte Lagoon, Tunisia). *Mar. Biol.*, 151, 767-783.

Stelmakh, L. & Georgieva, E. (2014). Microzooplankton: The trophic role and involvement in the phytoplankton loss and bloom-formation in the Black Sea. *Turk. J. Fish. Aquat. Sci.*, 14, 955-964.
